# Agriculture: science and technology safeguard sustainability

**DOI:** 10.1093/nsr/nwz036

**Published:** 2019-03-16

**Authors:** Hepeng Jia

**Affiliations:** Freelance science writer based in Ithaca, New York, USA and Beijing, China

## Abstract

China has traditionally placed tremendous importance on agricultural research. Meanwhile, in recent years, sustainable agriculture has been increasingly highlighted in both policy agenda and the capital market. However, while terms like environmental friendliness, low carbon, organic and green agriculture have become buzzwords in the media, few meaningful discussions have been raised to examine the relationship between science and technology (S&T) development and sustainable agriculture. What's more, some environmentalists stress that sustainable agriculture should abandon modern agriculture's heavy reliance on science and industrialization, making the link between agricultural S&T and sustainable agriculture seem problematic. What is the truth? If S&T are to play an important role in advancing sustainable agriculture, what is the current status of the field? What factors have caused the sustainable development of agriculture in China? At an online forum organized by *the National Science Review* (*NSR*), Hepeng Jia, commissioned by *NSR* executive editor-in-chief Mu-ming Poo, asked four scientists in the field to examine the dynamic relationship between sustainable agriculture and agricultural S&T in the Chinese context.

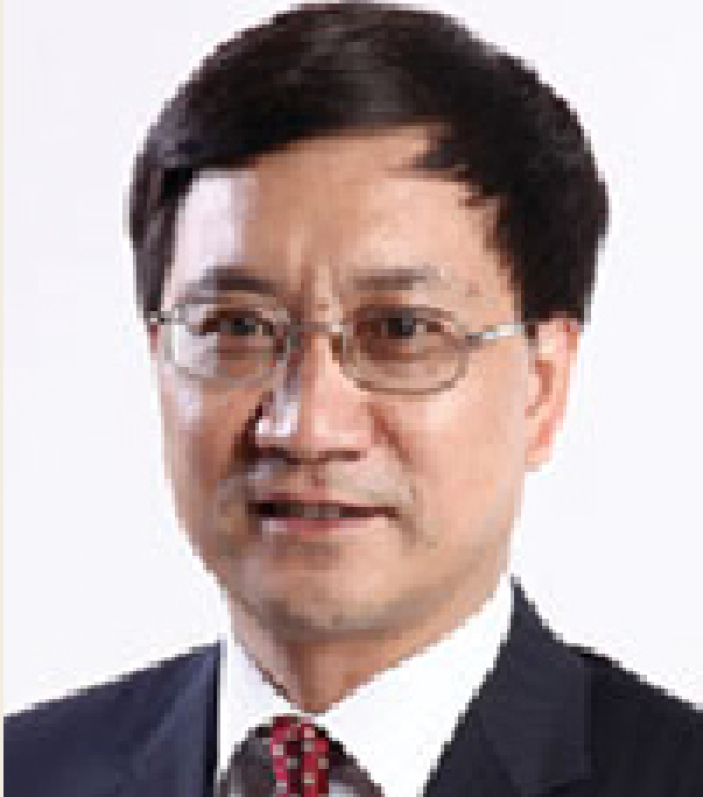

Jikun Huang

Agricultural economist at Peking University, Beijing, China

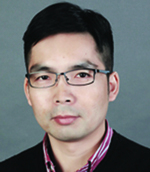

Xiaofeng Luo

Agricultural economist at Huazhong Agricultural University, Wuhan, China

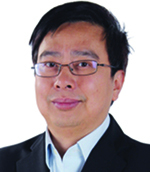

Jianzhong Yan

Agricultural and environmental scientist at Southwest University, Chongqing, China

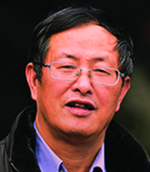

Yulong Yin

Veterinary scientist at Institute of Subtropical Agriculture, Chinese Academy of Sciences, Changsha, China

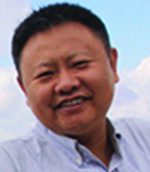

Hepeng Jia (Chair)

Science communication scholar at Cornell University, Ithaca, NY, USA

## PROPERLY DEFINING SUSTAINABLE AGRICULTURE

**Jia:** In recent years, sustainable agriculture has become a hot issue in China. Meanwhile, the term is often confused with organic or green agriculture. In 2015, the State Council issued a national outline on sustainable agriculture. I guess that there should already be an authoritative definition. Let's discuss this first.

**Luo:** I personally think that low-carbon agriculture, organic agriculture or other concepts have emphasized different aspects of sustainable agriculture. For example, low-carbon agriculture stresses its energy-efficiency. Organic agriculture emphasizes the controlled use of chemical fertilizers and additives. Sustainable agriculture, by comparison, lies at a higher and more comprehensive level.

**Yin:** I think the concept of sustainable agriculture means that it realizes the balance of supply of agricultural products for contemporary human beings without destroying the resources for and the interests of future generations. It is the long-term stable development of agriculture and resources, but the key is to apply modern science and technology (S&T) to solve the bottleneck problems that restrict the sustainable development of agriculture.

No matter whether this sustainable agriculture features low-carbon agriculture or organic agriculture, the most fundamental aspect of sustainable agriculture is to adopt modern technology.

—Yulong Yin

No matter whether this sustainable agriculture features low-carbon agriculture or organic agriculture, the most fundamental aspect of sustainable agriculture is to adopt modern technology. Only focusing on low-carbon agriculture or organic aspects of agriculture is not sustainable.

**Jia:** Prof. Yin said that technology plays a very important role in sustainable agriculture, but there are also some environmentalists who think that modern agriculture is too deeply affected by technology, which has impacted the sustainability of agriculture.

**Huang:** I agree with Prof. Luo and Prof. Yin. Sustainable agriculture does not only mean sustainability, but also means the growth of agriculture. Technological innovation is very important. Given the limit of water and land resources, you have to produce more products to meet food demand, you have to increase the productivity, and for certain products, the increased output must be achieved with fewer resources. Genetically modified (GM) crops are one of the many S&T innovations needed to support sustainable agriculture.

**Yan:** I am also interested in debates on the path of sustainable agriculture. In particular, there are some disputes between developed and developing countries on some aspects of sustainable development. Many developed countries have proposed sustainable development based on the situation of having abundant land resources. They adopt land- and capital-intensive patterns of agriculture and now they have more resources to stress environmental friendliness.

But in many developing countries, they have not come out of the so-called Malthusian trap [Editor's note: population outgrows resources and subsistence, leading to food shortages]. They still have poor people who struggle to make a living, are still destroying the environment and causing environmental pollution. Therefore, the understanding of sustainable agriculture between developed and developing countries may be different. China has some characteristics of developed countries and some characteristics of developing countries.

In the past two or three decades, China has experienced the process of the intensification of agriculture. In particular, China has been experiencing a revolution in agriculture, mainly characterized by the intensive use of labor force and capital in agriculture, for example, in vegetable farming. So, in this respect, we really have had some measures of intensive agricultural development as in developed countries.

On the other hand, the vast western part of China still has not got rid of the Malthusian trap. In these places, the problems of desertification and land loss are still very serious, and even more serious than in the past. So, in general, in terms of sustainable development, we have to adopt different paths to sustainable agriculture in different regions.

## SUSTAINABLE AGRICULTURE AND TECHNOLOGICAL DEVELOPMENT

**Jia:** The panelists have a strong consensus that sustainable agriculture cannot be separated from modern S&T. Now let's examine how S&T innovations can promote sustainable agriculture.

**Yin:** I believe that achieving sustainable development of agriculture must rely on technology. At the primary level, technology can improve agricultural output, solving the contradiction between huge food demands and limited amounts of farmland.

We consume a lot of meat every day. But meat production is constrained by China's lack of land. For example, we need around 200 million tons of feed per year in China, more than the USA. Over 60% of that is imported. I believe that traditional or organic agriculture cannot fundamentally maintain China's food security and the sustainable development of agricultural restructuring.

In the past 20 years, S&T progress in animal husbandry has increased the survival rate of baby animals by 30% and improved feed conversion by 30%, and reduced nitrogen and ammonia emission by 20%, water consumption by 10% and feces production by 15%. Therefore, modern S&T play a key role in improving animal husbandry's outputs, minimizing its consumption of arable land and water resources, and reducing its pollution emissions. This certainly has contributed to the sustainable agriculture development.

However, domestic livestock and poultry farming still suffer from problems such as low feed utilization rates and epidemic disease. These lead to the inefficient use of food resources, feed waste and environmental pollution.

So, what do we do? We have to rely on modern agricultural technology. We have to improve water utilization efficiency. We must combine the farming and breeding industries. Agricultural mechanization is also very, very important. Some of the young people in our village now go to cities to work, so we have to engage in intelligent agriculture. We must make our agricultural machinery highly efficient, so that we can make our agriculture sustainable.

**Luo:** In the development of the entire agricultural economy, the important role of S&T goes without saying. First of all, it can compensate for the lack of resources, whether it is land or water. If you have to feed so many people, you must rely on technology under the premise of this rare land or water. Second, technology can also improve the efficiency of our agriculture. Traditionally, food security is the pursuit of the grain output. The reason why we have achieved continued grain output growth in these years is technology improvement.

For sustainable agriculture, China has some characteristics of developed countries and some characteristics of developing countries.

—Jianzhong Yan

Then, I think technology is also improving our ecological environment. In recent years, China's agricultural ecological environment has been improved. This is, in large part, because technology plays a role, for example, reducing unnecessary resource consumption in agriculture.

Finally, agriculture is not the same as other industries. It is easily subject to natural disasters. There are many such unpredictable natural disasters. To reduce this risk, it is very important to rely on technology.

**Huang:** I would also add a little bit. Many technologies can promote the sustainable development of agriculture. Now irrigation technology, including water-saving irrigation technology, is very important. Chemical technology innovation promotes the uses of quality fertilizers and low-toxicity pesticides in China. Various bio-pesticides as well as the biological control of pests will play an important role in our sustainable development.

There are also some big improvements in agronomy, including the systematic integration of farming and animal husbandry. The application of ICTs (information and communication technologies) in agriculture is also emerging. They will have great impact on precision agriculture and more efficient use of resources. I think that the most important thing to talk about is biotechnology, because biotechnology is one of the most important technologies for promoting agricultural development, especially for sustainable agricultural development.

## ADVANCING GOOD POLICIES FOR SUSTAINABLE AGRICULTURE

**Jia:** Sustainable agriculture needs a good system to support. Let's explore this aspect further.

**Huang:** Science and technology innovations are very important to sustainable agriculture, but we need a good incentive system and favorable institutional arrangements for these innovations. We have to provide better incentives for the scientists in the innovations, and appropriate incentives for farmers to use new technologies in agriculture. Generating new technology needs institutional guarantees. If you do not have a good national institutional arrangement, it is also difficult to generate and commercialize innovative technologies.

**Figure:**
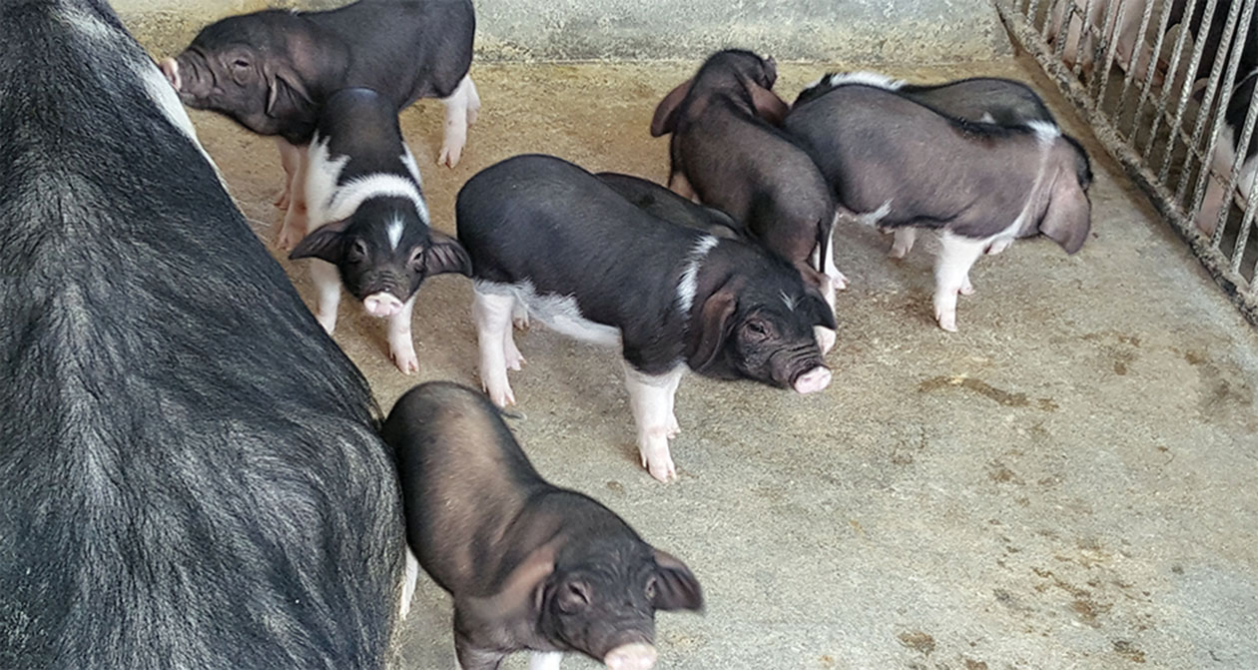
A pig farm in China as seen from outside. Facing challenges ranging from diseases like African swine fever to improving meat quality, China's animal husbandry is in urgent need of adopting modern S&T to support its sustainable development (*Courtesy of Yulong Yin*).

**Jia:** We all know that, in 2015, the State Council released a long-term plan for sustainable agriculture (2015–2030). Why is this important? Why do we need a State Council regulation rather than simply a ministry order? How can policies promote technology development for sustainable agriculture?

Science and technology innovations are very important to sustainable agriculture, but we need a good incentive system and favorable institutional arrangements for these innovations.

—Jikun Huang

**Huang:** The development of sustainable agriculture has gone beyond any individual ministries. It is not simply about the environment, agricultural production or agriculture S&T. Therefore, top-level policy design and coordination are necessary.

**Yin:** I participated in some discussions about the development plan. It involves national food security, financial security and ecological security issues. The agricultural sector alone cannot be relied upon, nor is it completely implemented by the planning departments, nor is it solely done by the environmental agencies. The involvement of National Development and Reform Commissions (NDRC), the Ministry of Finance and the Ministry of Education are all essential. To solve sustainable developments in agriculture, we must achieve the goals set by our agricultural S&T development plan. It needs top-level design and institutional arrangements at the national level so that all agencies can participate.

Then I think the plan enacted by the State Council means that its significance is very high, especially in the background of implementing the Beautiful China task required by President Xi Jinping. When we build a beautiful China, we must do this with sustainable development of agriculture. This is the new agricultural modernization road with Chinese characteristics. Top-level policy design and implementation are a must.

Meanwhile, while China's agricultural and rural economy has made great achievements, we also face some problems, that is, the excessive development of rural resources, the high input in agricultural production, and the excessive use of natural resources, especially groundwater. Therefore, the State Council issued such a document. It is a programmatic outline and an agenda of action.

**Luo:** The State Council's development plan is not just one document, but it also includes the Beautiful China strategy mentioned by Prof. Yin, which involves many ministries. So, if we want to promote sustainable development of agriculture, we need to have institutional arrangements. This national agricultural sustainable development plan is a type of institutional arrangement. Second, because the situations in different regions are different, the regional sustainable development strategies are definitely not the same.

## FROM THE HOUSEHOLD RESPONSIBILITY SYSTEM TO SUSTAINABLE AGRICULTURE

**Jia:** We have discussed institutional arrangements for sustainable agriculture. China's household responsibility system for farmland use [Editor's note: the system enables Chinese peasants to hold long-term farmland-use rights for decades or even longer without legally changing the literal collective land ownership] has resulted in small-scale family farms, which may result in short-sighted behavior in agriculture. Will such short-sighted behavior impact sustainable agriculture?

**Huang:** The household responsibility system is a basic institutional arrangement in rural China and was the greatest institutional innovation in the past. I do not agree that it resulted in short-sighted behavior. This institutional reform has provided great incentives for farmers to raise productivity and increase agricultural production. Of course, it also leads to small-scale farming systems. But there are also many other institutional innovations that have promoted land consolidation, such as the land transfer platforms and land rental markets. In fact, the usage rights of more than one-third of farmers’ contracted land are now being transferred between farmers.

China does not have the natural conditions to develop big farms as in North and South America, but the scale of land transfers is not low. Let's compare with our neighbors. Japan and South Korea have adopted private ownership of land. But their land transfers in the past century were not higher than China in the past one or two decades. So, the key is not the scale of farms but developing appropriate farm sizes, generating advanced technologies that these farms can use, and offering off-farm jobs to rural laborers so that farm sizes will expand and farmers’ incomes will rise.

**Yan:** I will add one more point. In the past few years, there has been a lot of comparative research in China, comparing these small family farms with large farms set up on the basis of land transfer. It was found that in Shandong Province's vegetable or tobacco planting, small family farms have higher net returns and more productivity. The reason is simple. Large farms with transferred farmland pay rent and labor costs at the price of non-agricultural workers, but small family-run farms use their own elderly and women. They don’t need to pay wages, so this small farm is completely capable of competing with the ranch.

In fact, we are currently doing a lot of investigations in Chongqing and other southwestern regions. All the farms with transferred land are losing money. After the loss, they have to find local governments to provide them with subsidies. With government subsidies, some big farm operators insisted on operating their farms but made more losses. These facts show that land transfer and the corresponding large farms are not certainly the answer to sustainable agriculture.

China does not have the land resources to encourage large-scale farms like in the USA. Then, should labor- and capital-intensive farms of appropriate sizes be the main direction of our agricultural development?

**Jia:** Dr Yan has raised an important aspect of China's modern agriculture, the labor- and capital-intensive middle-sized and small farms. Can we elaborate on this in the context of sustainable agriculture?

**Yan:** This kind of new agriculture now accounts for about one-third of the cultivated land, but its output value is very high.

Conventional agriculture accounts for two-thirds of the country's cultivated land, planting crops such as grain, cotton, and rape, but its output value only accounts for over 10%. We will further pursue this labor- and capital-intensive agriculture in the future. This is because Chinese people have changed their food consumption structure from dominantly relying on grains to consuming a high amount of meat, eggs, vegetables and fruits. This is a natural result of our higher income. This has created good opportunities for sustainable agriculture.

The increasing number of middle-class people and their higher incomes have resulted in a huge demand for high-quality organic products. We do not talk about their production amount but their output value, because the current unit price of organic products is 10 times the unit price of conventional agricultural produce.

In fact, some regions in Shandong Province have already exported this organic agricultural produce to Japan and South Korea. Their tests are very strict. It is said that some of Shandong's organic agricultural produce exports to Japan will undergo tests with more than 600 components. This type of labor- and capital-intensive small farm is transforming our agriculture. Because we now have so many middle-class people, meeting their demands will promote the transformation of agriculture into these high-value small farms.

**Yin:** I have a slightly different view. The household responsibility system has its own limitations, such as hindering the development of agricultural mechanization. If I only have two or three mu (1 mu = 0.16 acre) of land in my household, how can we engage in agricultural mechanization? This small farm may waste the production resources and the productivity is low.

We have been discussing land transfer. But in my hometown, much farmland is no longer planted. All the young peasants are working in cities. The land is deserted there. But in some cases, when the land can be transferred to be concentrated with one or two very capable farmers, they have incentives to cultivate the farmland, as the land concentration will not only lower costs but can also be used for finance.

## FROM UTILIZING MECHANIZATION TO OVERCOMING INSUFFICIENT AGRICULTURAL LABOR

**Jia:** Prof. Yin has raised an important point – the abandonment of farmland. In fact, it is inevitable to talk about China's urbanization here. After working in cities, young peasants will not return to the countryside. Will this have a great impact on sustainable agriculture?

**Luo:** Agricultural development needs a high-quality workforce. Currently, the rural labor force dramatically flows to the city, leaving the countryside with old people. I think we may need technology to solve insufficient labor quantity and quality.

At present, many regions are promoting the use of technologies, such as drones or information technology, to solve the problem of insufficient labor. The development of this smart agriculture can be a very important direction for our sustainable agriculture.

**Yan:** At present, the ways to solve labor outflows in different regions vary. Social services, such as providing machine sowing, machine harvesting, or socialization, are adopted to solve the problem in many parts of China. At present, the demand for labor in plains areas is greatly reduced, and seniors who remain in the countryside can meet the demand.

But in mountainous areas, the problem is very obvious. The cultivated land is far away from the residents. Therefore, in mountainous areas, farmland abandonment is particularly serious. But there are also mechanization efforts to overcome this. Small farms may hinder the application of mechanization. But in recent years in China, small and micro machines, such as handheld grass cutters and micro tractors, have been widely used. This can solve the labor insufficiency after young peasants move to cities.

In some areas where using machines is too difficult, we can simply abandon them, and they can be reforested. Abandoning marginal land is a worldwide trend. There is nothing to worry about. In fact, despite workforce loss and abandoned land, China's agricultural outputs have kept on growing in recent years.

**Huang:** I want to follow up the point on outflow of young labor from the countryside. This is a reality but certainly not a problem. The average age of agricultural labor in China is about 53 to 54. In the USA, the average age of farmers was 58.3 in 2012 while, in Japan, the average age is 67. This is a result of natural selection or division of labor. Senior persons generally have more comparative advantage in farming than in manufacturing or service industries, while youths have less comparative advantage in farming than other sectors. In most countries, you will find a positive association between per capita income and the age of agricultural labor.

So, the problem is not that we have older peasants left in rural areas, but how to improve their capacity to use new technologies. Don’t expect many young peasants to return to the countryside for farming.

Technological innovation can help to deal with the ageing issue in farming. For example, agricultural machines can be made easier to use for seniors.

We need technology to solve insufficient labor quantity and quality needed for sustainable agriculture.

—Xiaofeng Luo

**Figure:**
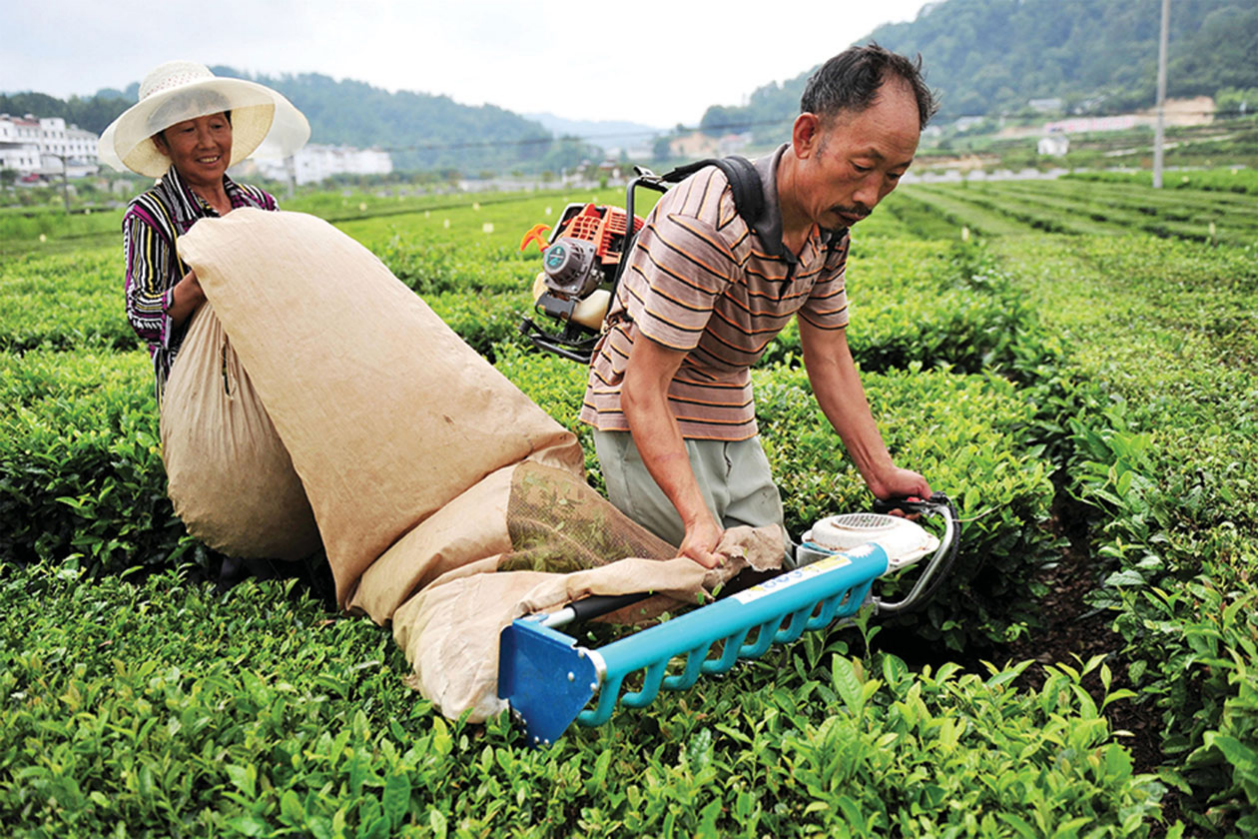
A tea farmer in the Chinese province of Hubei picking tea with a tea-picking machine. Small-scale machinery has been widely used in the Chinese countryside to solve labor shortages while improving the efficiency of sustainable agriculture (*Source: China News Service Photo*).

Another point is the difficulty in mechanization for farmland in mountain areas. While small machines can partially help to solve this problem, with higher labor costs, planting conventional crops in these areas may not have reasonable profit. I think this can be partially solved by transforming production structure from the current crops to forage grass or orchard production. In many southern mountainous areas, natural conditions are very suitable for developing grass.

**Yin:** Growing herbal plants may be considered as one division of these grass and timber industries. In recent years, it has developed very quickly in my hometown province of Hunan.

**Yan:** The central government now has subsidized agricultural mechanization, including using micro machines in mountainous farmland. Now rechargeable grass cutters are widely used in the Chinese countryside. So here we have another example of S&T innovations promoting sustainable agriculture.

## FARM TRADE AND SUSTAINABLE AGRICULTURE

**Jia:** Like the outflow of young agricultural labor, another major trend in agriculture in China is the massive imports of agricultural products such as soybean and corn. How will the farm trade impact on sustainable agriculture?

**Huang:** I want to correct one point. We are the world's largest importer of agricultural products in terms of total amounts, but China's per capita import values are one of the lowest. China imports land- and water-resource-intensive agricultural products but exports products like fruit, vegetables, fish and processed food products. The net import is only about 5% of our total food consumption, primarily soybean, sugar and dairy products. If counted in water, the amount of water needed to irrigate the imported agricultural products in 2015 was equivalent to 25% of the total irrigation water in China in that year. If counted in land, the amount of land needed to plant the imported agricultural products in 2015 was equivalent to 35% of China's total farmland. Therefore, imports substantially reduce China's pressure on scarce land and water resources, contributing to sustainable agriculture.

**Yin:** I basically agree with Prof. Huang. But facing the current trade war, it is necessary for China to increase its own soybean output, so that our food security can be safeguarded.

**Yan:** What Prof. Huang recommended is equivalent to the so-called virtual water and virtual land. With the globalization of resources and supply chains, it is reasonable for the government to study how to ‘save’ and ‘develop’ these virtual resources. For example, African and South American countries are also very eager to sell their agricultural products to us. Carefully planning these virtual resources will promote China's sustainable agriculture.

## BIOTECHNOLOGY AND SUSTAINABLE AGRICULTURE

**Jia:** This forum was scheduled to discuss the role of agricultural biotechnology and particularly GM (genetic modification) technology in sustainable agriculture. Prof. Huang is the nation's top expert on this. Can you clarify the relationship?

**Huang**: GM technology has been shown to increase crop yield and lower production costs, which raises farmers’ income. With the adoption of GM technology, China can also reduce its agricultural imports. With rising agricultural production and therefore falling prices, the competitiveness of China's agriculture can be improved, which contributes to China's national food security. Because most of the current GM technologies are resistant to insects, the technologies have significantly reduced the amount of pesticides used. The biggest problem with food security now is the high residue of pesticide in food, and the development of our GM technology can promote food security by reducing pesticides.

GM technology can also improve the efficiency of all chemical use. By reducing the use of pesticides and fertilizers, GM technology will have a very important impact on our greenhouse gas emissions and mitigate the impacts of climate change.

In addition, the drought-resistant GM varieties can save water. Taken together with GM technology, we will be able to develop more resource-saving agriculture, and the increase in productivity is equivalent to saving farmland for a given output.

In fact, agricultural biotechnology is not limited to GM technology. It is estimated that the annual output value of agricultural biotechnology should have surpassed 100 billion yuan (US$14.4 billion), including animal biotech medicine, animal vaccines, biotech fertilizers, molecular breeding, ecological protection and so on. In addition to GM technologies, there are many new types of agricultural biotechnology, such as new enzymes that transform straw to feed suitable for ruminant animals through fermentation. Using corn to feed pigs, perhaps products from 1 mu of corn can only meet the food demands of one pig, but if we can transform straw to feed, we can raise five pigs using the same amount of farmland.

**Jia:** Given the vital importance of agricultural biotechnology, particularly GM technologies, in sustainable agriculture, why did the abovementioned 2015 national plan on sustainable agriculture not mention GM technologies?

**Huang:** One reason could be planning officials’ lack of knowledge on GM technologies. But more importantly, it might be influenced by public opposition. Our studies show that, in 2001, the percentage of consumers accepting GM technologies reached two-thirds, but it declined to 24% in 2012, and further down to just over 10% in 2016. More science popularization efforts for GM technologies should be made, but on the other hand, policymakers should not rely too much on public opinion to decide whether to advance GM technologies. There should be political commitment to push ahead and commercialize GM technologies given their tremendous role in raising agricultural productivity and sustainable development of agriculture.

## FACING CLIMATE CHANGE CHALLENGES

**Jia:** Climate change is one of the grand challenges that human beings face. What can sustainable agriculture do for us in this aspect?

**Yan:** We have been studying the impact of climate change in the Qinghai–Tibet Plateau. Well, climate change may have some positive impacts on China's agriculture, and it may also have negative impacts. As the climate warms, it makes the planting of many crops in China move northward and often westward, right? In the past few years, the expansion of many crops such as glutinous rice and wheat has been very obvious.

On the other hand, the negative effects of this climate change are that there are more disasters. Many of these droughts, and droughts caused by this extreme climate in particular, have a great impact on pastoral areas! In other words, climate warming will exacerbate the prevalence of pests and diseases, making the use of pesticides increase. In addition, higher temperatures have also caused soil to lose organic matter, which has accelerated the degradation of the soil. Both the positive and negative impacts of climate change raise challenges to sustainable agriculture.

**Huang:** I agree with Dr Yan. Climate change has different effects in different regions in China. So simply talking about how climate change is a problem for sustainable agriculture is not enough. It has both positive and negative influences. In particular, there are management issues related to water, because no matter whether the consequence of climate change is drought or floods, they are related to water management. Therefore, we should further strengthen the utilization of water resources, improve their efficient use, and manage related issues in water resources. This is an important aspect of sustainable agriculture.

Just now we emphasized the role of technology in sustainable agriculture. Faced with the challenge brought by climate change, it is necessary to promote sustainable agriculture with technology.

